# Tracing the emergence of multidrug-resistant *Acinetobacter baumannii* in a Taiwanese hospital by evaluating the presence of integron gene *intI1*

**DOI:** 10.1186/1477-5751-13-15

**Published:** 2014-08-14

**Authors:** Chang-Hua Chen, Chieh-Chen Huang

**Affiliations:** 1 Division of Infectious Diseases, Department of Internal Medicine, Changhua Christian Hospital, Changhua 500, Taiwan; 2 Infection Control Committee, Department of Internal Medicine, Changhua Christian Hospital, Changhua 500, Taiwan; 3 Department of Nursing, College of Medicine & Nursing, Hung Kuang University, No. 1018,Sec. 6, Taiwan Boulevard, Shalu District, Taichung 43302, Taiwan; 4 College of Life Science, National Chung Hsing University, 250 Kuo-Kuang Road, Taichung 402, Taiwan

**Keywords:** *Acinetobacter baumannii*, *IntI1* gene, Class 1 integron

## Abstract

**Background:**

In Changhua County, Taiwan, the number of clinical *Acinetobacter baumannii* isolates has risen since 2002, and multidrug-resistant *Acinetobacter baumannii* (MDRAB) has spread rapidly throughout Taiwan. In this study, to reveal the mechanism involved with the rapid dissemination of MDRAB emergence, the utility of the class 1 integron, *intI1* integrase gene, as an MDRAB-associated biomarker was examined. A cross-sectional, clinical epidemiological study was performed at Changhua Christian Hospital between January 1^st,^ 2001 and December 31^st^, 2004. Besides the existence of *intI1* gene was examined, the pulse-field gel electrophoresis (PFGE) was also performed to determine the epidemiological characteristics of the isolates.

**Findings:**

The overall hospital infection rate was 5–6%, while the infection rate of the intensive care unit (ICU) fluctuated. No positive correlation was observed between MDRAB isolates and the presence of *intI1* (r = 0.168, *P* = 0.254). Additionally, no positive correlation was observed between the infection rate in the ICU and the presence of *intI1* (r = -0.107, *P* = 0.468) or between the hospital infection rate and the presence of *intI1* (r = -0.189, *P* = 0.199). However, two predominant clones among the MDRAB isolates were identified by PFGE.

**Conclusions:**

Although the presence of the *intI1* gene does not seem suitable for tracing MDRAB emergence in Changhua County, two predominant clones were identified by PFGE, and subsequent studies to identify whether these clones were responsible for original nosocomial infection are needed.

## Findings

### Introduction

Multidrug-resistant *Acinetobacter baumannii* (MDRAB) is globally recognized as an important cause of nosocomial infections during this century [1]. Both environmental adaptation and rapid acquisition of resistance genes have contributed to the ability of *Acinetobacter baumannii* (AB) to be such a prosperous pathogen [2]. These resistance genes are primarily spread through horizontal gene transfer (HGT) of plasmids, transposons, or integrons that carry gene clusters that provide resistance to several antibiotic families [3]. Resistance islands (RIs) are important elements for acquiring foreign genes by HGT [4]. The RIs containing the integrons have been reported in many MDRAB strains [5-8]. Within Changhua County (CC), Taiwan, the numbers of clinical AB isolates have risen since 2002, and the first MDRAB was reported in Taiwan that same year [9]. Since then, MDRAB has rapidly spread through Taiwan and been an emerging problem. Though early detection is critical for isolation and targeted therapy, no well-known biomarker for detecting those mobile characteristics exists because of the structural diversity of RIs of AB. On the other hand, the class 1 integron-carrying MDRAB clones were reported to be widespread in Taiwan [10], while Koeleman showed that amplification of the *IntI* gene may act as a rapid and simple technique for the routine screening and identification of clinical *A. baumannii* isolates with epidemic potential [11]. Another study showed that integron detection could be a useful tool for studying molecular epidemiology in hospital environments, facilitating the quick detection of possible cross-infection cases [12], especially in critical wards such as the intensive care unit (ICU). To reveal the mechanism involved with the rapid dissemination of MDRAB emergence, the presence of the *intI1* integrase gene in class 1 integrons was assessed as an MDRAB-associated biomarker in this study.

## Methods

### Clinical investigations

The Changhua Christian Hospital (CCH) is an 1800-bed tertiary teaching hospital located at CC, Central Taiwan. A cross-sectional, epidemiological study of MDRAB isolates was conducted at CCH from January 1^st^, 2001 to December 31^st^, 2004. Infectious disease specialists reviewed all patient medical records. Because and our findings disclosed MDRAB*,* which were resistant to many broad-spectrum antibiotics (including ciprofloxacin, piperacillin-tazobactam, ceftazidime, cefepime, imipenem-cilastatin, amikacin) since 2001–2002 and we reviewed literature (Additional file 1), the critical period for the appearance and spread of MDRAB was estimated to be 2001–2002. We choose the study year is 2001–2002 with the hypothesis of study year is the beginning of the increment of resistant *A. baumannii* infection. Concerning the observation year, we selected the 2003–2004 for comparison. If the hypothesis is correct, the presence of the *intI1* integrase gene in class 1 integrons will increase between the study year and the observation year. The Standard Centers for Disease Control and Prevention National Nosocomial Infections Surveillance definitions for nosocomial infections were used [13]. The hospital infection rate and the ICU infection rate were calculated. We also performed retrospective surveillance for nosocomial infections in the ICUs.

### Microbiological investigations

All AB isolates were routinely examined at Medical Laboratory Department of CCH and stored in the Bacterial Bank of CCH from January 1^st^, 2001 to December 31^st^, 2004. Phenotypic methods were used to identify *A. baumannii* isolates using a Vitek-2 System (BioMerieux, Marcy l'Etoile, France). Additionally, antimicrobial susceptibility was determined using an automated Vitek 2 (bioMe'rieux, Marcy l'Étoile, France) according to the recommendations of Clinical and Laboratory Standards Institute [14]. In this study, MDRAB was defined as resistance to at least two specific representatives of at least two classes of antibiotics among those antibiotic categories, including aminoglycosides, anti-pseudomonal penicillins, carbapenems, third or fourth generation cephalosporins and quinolones [15].

### Molecular detection of *IntI1* integrase gene

As previously described, the Koeleman’s method was used to amplify the targeted *intI1* gene [11]. We used random sampling methods for the retrospective analysis during January 1^st^, 2001 to December 31^st^, 2004, and a half of clinical MDRAB isolates monthly were selected, but no more than 10 isolates among each months, during the study period. Overall 440 MDRAB isolates were tested for the target gene intI1 during the study period.

### Pulse-field gel electrophoresis (PFGE) analysis

The ampicillin-sulbactam resistance phenotype, *IntI1* integrase positive genotype, and strains isolated within season of high infection rate were chosen as the selection criteria for listing candidates MDRAB strains. Standard protocol for PFGE analysis was employed for the *A. baumannii* isolates. Briefly *A. baumannii* were plated on blood agar and incubated in a 5% CO_2_ atmosphere at 35°C for 16–24 h. Plug slices were digested with 20 U of *Sgr*AI. The DNA fragments were then separated in 1% Seakem Gold agarose gels (FMC BioProducts) at 14°C using a Bio-Rad CHEF DRIII PFGE system (Bio-Rad Laboratories, Hercules, CA, USA). Gels were run in 0.5× Tris-borate-EDTA (TBE; pH 8) at a 120° fixed angle and a fixed voltage (6 V/cm), with pulse intervals from 4–40 s for 20 h. Following staining and imaging, the chromosomal DNA restriction patterns produced by PFGE were interpreted using Tenover’s categorization [16].

### Statistical analysis

We used the finite mixture model on categorical dataset of antibiotic resistance phenotypes. The results were considered significant when the *P* value was less than 0.05. All data were analyzed using a SPSS software v10.0. We used the χ^2^ test on categorical data to analyze relations. We used the Spearman’s correlation to analyze the relations between different parameters.

## Results

During the past fifteen years, the overall hospital infection rate at CCH was 5-6%, and the infection rate in the ICU fluctuated. During this time period, patients with *AB* infections increased gradually at CCH, especially in the ICU (data not shown). Between January 1^st^, 2001 and December 31^st^, 2004, two periods occurred during which the number of AB*-*infected patients increased. The Infection Control Office of CCH began to investigate the infection rate in the hospital and the ICU (Additional file 2). Several periods occurred during which the infection rate in the ICU increased, including June 2001, November 2001, February 2002, July 2002, September 2002, July 2003, November 2003, and March 2004. However, the reason for the gradual increase in *AB* infection is still unknown, despite investigation. No significant changes in the numbers of MDRAB isolates and the carrying rate of *IntI1* gene were observed when the data from 2001–2002 was compared to that of 2003–2004.

Using a retrospective analysis and Koeleman’s method, the results of amplifying the *IntI1* gene from clinical 440 MDRAB isolates are shown in Table 1. No positive correlation was observed between clinical MDRAB isolates and the presence of the *IntI1* in the bacteria (r = 0.168, *P* = 0.254). Additionally, no positive correlation was observed between the infection rate in the ICU and the presence of *IntI1* (r = -0.107, *P* = 0.468). Furthermore, no positive correlation was observed between the rate of hospital infection and the presence of integron (r = -0.189, *P* = 0.199). Thus, the presence of *IntI1* integrase gene does not correlate with MDRAB prevalence and spread during the study period.

**Table 1:** **Epidemiological features of the ICU infection rate, hospital infection rate, clinical multidrug-resistant *A. baumannii *** **isolate numbers and *IntI1*-containing rate during the 2001 and 2004**

**Year**	**Carrying-*Int I1*** **rate**	**MDRAB isolates number**	**ICU infection rate**	**Hospital infection rate**
2001	r	0.080	-0.235	-0.586
	p-value	0.805	0.462	0.045
2002	r	0.055	-0.088	-0.228
	p-value	0.864	0.785	0.476
2003	r	0.594	0.041	0.305
	p-value	0.042	0.899	0.334
2004	r	-0.086	-0.172	-0.135
	p-value	0.790	0.594	0.675
Overall	r	0.168	-0.107	-0.189
	p-value	0.254	0.468	0.199

Notes: *r*: Spearman’s Correlation Coefficient, *MDRAB*: Multi-drug resistant A*. baumannii.*

To evaluate the epidemiological features of the MDRAB isolates, selection criteria such as ampicillin-sulbactam resistance phenotype, *IntI1* integrase positive genotype, and strains isolated within season of high infection rate were used for choosing candidates MDRAB strains (Table 2) Eight of the clinical MDRAB isolates (two isolates each year), which carried *IntI1* positive genotype and ampicillin-sulbactam resistance phenotypes were chosen in each July during the four study years. And, the other four clinical MDRAB isolates (one isolate each year), which is resistant to ampicillin-sulbactam but without carrying *IntI1* gene, were also employed as reference strains during the same study period. The interpretation method for PFGE results is according to Tenover’s criteria [16], and the statistic method is descriptive method (Table 2). Though PFGE results identified two predominant clones types (clone A, B) during the study year (Table 2, Additional file 3), the relationships between the predominant clones and the presence of the *IntI1* did not have a significant association.

**Table 2:** **Summary for results of PFGE fingerprint patterns and antibiotic susceptibility among clinical *Acinetobacter baumannii *** **isolates in the PFGE study**

**Number**	**P1**^**1**^	**P2**	**P3**	**P4**	**P5**	**P6**	**P7**	**P8**	**P9**	**P10**	**P11**	**P12**
**Isolates year**	**2001**	**2001**	**2002**	**2002**	**2003**	**2003**	**2004**	**2004**	**2001**	**2002**	**2003**	**2004**
PFGE type	A	B	C	B	A	A	B	D	A	A	E	A
Carrying-*Int I1*	+	+	+	+	+	+	+	+	-	-	-	-
AN	R^2^	R	R	R	R	S	R	R	R	S	R	S
SAM	R	R	R	R	R	R	R	R	R	R	R	R
CTZ	S	S	R	S	R	S	R	S	R	S	S	R
LVF	S	S	S	S	S	S	S	S	S	S	S	S
IMP	S	S	S	S	S	S	R	S	S	S	R	S
PIP-TAZ	S	R	S	R	S	R	S	R	S	R	S	S
CRO	S	S	R	S	R	S	R	S	R	S	S	R

Notes:

^1^P number is the Lane number of PFGE study.

^2^S: Susceptible; R: Resistant; I: Belonged to resistant.

^3^The susceptibility tests were performed using the Vitek-2 GN card (Biomerieux, Marcy l'Etoile, France). The results were interpreted using the CLSI breakpoints (Clinical and Laboratory Standards Institute Performance Standards for Antimicrobial Susceptibility testing).

^4^Abbreviation AN: amikacin; SAM: Ampicillin-sulbactam; CTZ: Ceftazidime; LVF: Levofloxacin; IMP: Imipenem-cilastatin; PIP-TAZ: Piperacillin-tazobactam; CRO: Ceftriaxone; colistin and tigecycline and polymyxin B were not provided by Vitek-2 System.

## Discussion

This study is the first epidemiological study to evaluate the involvement of class 1 integron for the appearance and spread of MDRAB in central Taiwan. MDRAB has been reported in Taiwan since the early 21^st^ century [17], and we also disclosed the same trend of MDRAB at the CCH. After evidence-based literature review (Additional file 1), 2001–2002 was identified as the critical time period for the appearance and spread of MDRAB, so we engaged this study. Because of the structural diversity of RIs in AB, detecting these huge mobile elements is not simple. The epidemic class 1 integron-carrying MDRAB clone was reported in Taiwan [10]. Detection of this class 1 integron, which is located in an RI of AB, is convenient. To reveal the mechanism and to trace the rapid dissemination during the emergence of MDRAB, we choose the *IntI1* integrase gene as the target for detection. However, the presence of *IntI1* does not correlate with the emergence and spread of MDRAB in this study. The *IntI1* gene is present in 81.3% of clinical *AB* isolates at our institute [9] and 66.7% at Zhong’s study [18].

In this study, we reported that the presence of *IntI1* does not correlate with the emergence and spread of MDRAB, and it’s different from previous study [19]. The reasons for no correlation between the *intl1* gene and MDRAB could be multiple factors in this study. Firstly, since the structural complexity of RIs, the *IntI1* gene may be not a good marker to trace the appearance and spread of MDRAB prior to the dissemination of class 1 integron-carrying MDRAB clones. Secondly, *IntI1* is too common to be an accurate biological marker. The contributions of class 1 integrons in MDRAB involve their carried antibiotic cassettes. So, the presence of *IntI1* integrase gene along does not correlate with the emergence and spread of MDRAB in this study. Further examinations were needed to reveal the correlation between their antibiotic cassettes and emergence of MDRAB.

**Figure 1:**
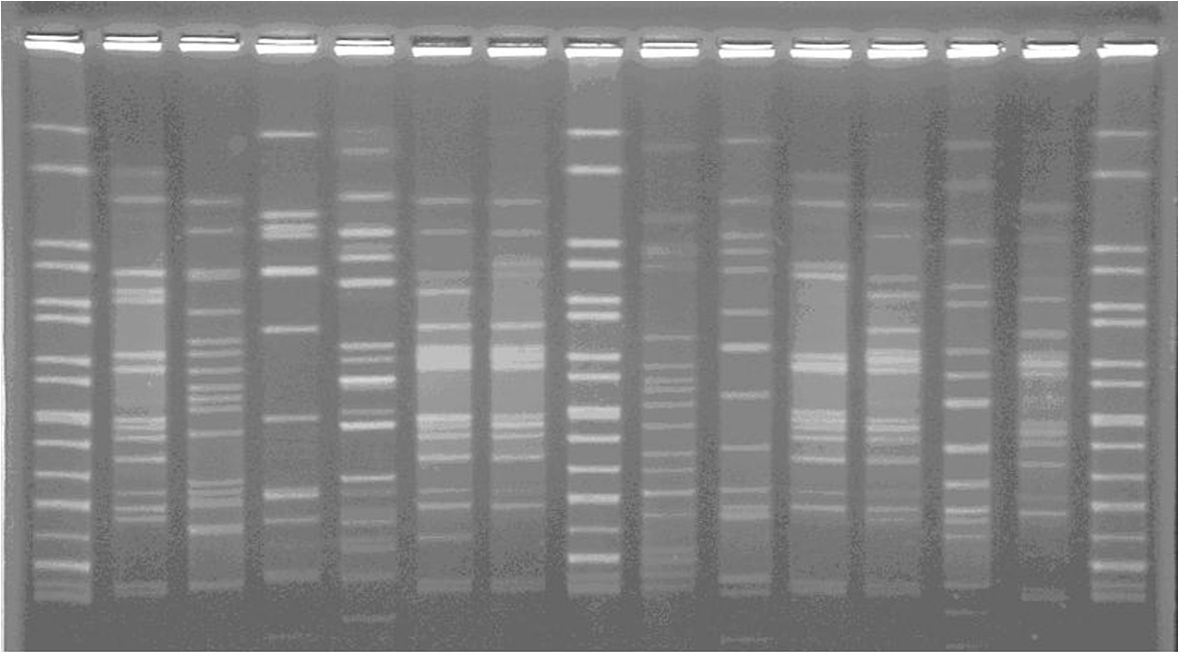
PFGE fingerprints of 12 multi-resistant A. baumannii (MDRAB) isolates with resistance to ampicillin-sulbactam.

The susceptibility rates of sulbactam-containing combinations (such as ampicillin-sulbactam) for AB were 90.4–92.7% in Brauers’s study [20], but the resistance rate of sulbactam-containing combinations (such as ampicillin-sulbactam) for AB was 57.4-64.1% in Taiwan Surveillance of Antimicrobial Resistance program [21]. The resistant rates to piperacillin/tazobactam and third geenration cephalosporines and fluoroquinolones for MDRAB strains in Taiwan were around 75%, while the resistant rates to ampicillin-sulbactam for MDRAB strains in Taiwan was only 50% [21]. Since sulbactam resistance phenotpye of AB was shown to be unique in Taiwanese isolates, the resistance phenotpye of ampicillin-sulbactam was thus chosen as selection criteria for PFGE analysis candidates. With regard to genotyping for AB, PFGE, multilocus sequence typing, and rep-PCR have been used in previous studies [22-24], while PFGE is still considered as the common standard for typing the outbreak-related isolates of AB [19]. In this study, PFGE results identified two predominant clones (clone A, B) (Figure 1) during the epidemic season. The result supports that PFGE is more accurate than *IntI1*-PCR to differentiate MDRAB isolates in central Taiwan. In this study, the reasons to choose three kinds of selection marker are as following: The first one is ampicillin/sulbactam resistant phenotype. Ampicillin/sulbactam has been proven to be more efficacious than polymyxins in treating carbapenem-resistant *A. baumannii* infection [25]. Although resistance to ampicillin/sulbactam in *A. baumannii* has been reported in many countries [1] and authors disclosed one unique phenomenon is that some isolates of MDRAB, which were resistant to many broad-spectrum antibiotics (including ciprofloxacin, piperacillin-tazobactam, ceftazidime, cefepime, imipenem-cilastatin, amikacin) but susceptible to ampicillin/sulbactam between 2001 and 2002 [9]. No resistance mechanism was described in those studies. The second marker is *intI1* integrase gene in class 1 integrons, and that is the target gene in this study. The last marker is timeline with recrudescent tendency. Because of a lot of clinical *A. baumannii* isolates, we needed to select the important isolates to study. According to the Additional file 2 (which shows the infection rate of the hospital and the ICU during 2001–2004), the strains isolated within season of high infection rate are from summer season during the four study period. It would be interesting to know how many of the nosocomial or healthcare-associated MDRAB isolates belongs to the same fingerprint type, and we should be cautious to those predominant clones, which spreading among hospitals and disseminating their integrons involved gene cassettes. Further examinations are needed to reveal the facts.

Concerning infection rate of the hospital and the ICU, it was also examined in this study (Table 1, Additional file 2). Several periods occurred during which the infection rate in the ICU increased, including June 2001, November 2001, February 2002, July 2002, September 2002, July 2003, November 2003, and March 2004. New staff has begun to work after orientation and training in June and September since 1990 at our institute. Additionally, budget cuts resulted in downsizing and changes in staffing, and many buildings were erected at our institute. Thus, further study is needed to elucidate the reasons and factors for the increase in clinical MDRAB isolates at central Taiwan at the period of the early 21^st^ century.

## Conclusion

In this study, the emergence of MDRAB in a Taiwanese hospital was traced by the correlated between detection of *IntI1* and existence of MDRAB. This is the first cross-sectional, clinical epidemiological study on the involvement of class 1 integron in the emergence and spread of MDRAB in Central Taiwan. Though the existence of *intI1* gene is not suitable for tracing MDRAB emergence that occurred at CCH, two predominant clones were identified from PFGE study, and subsequent studies to determine whether these clones are responsible for the original nosocomial infection are needed.

## Abbreviations

AB: *Acinetobacter baumannii*; CC: Changhua County; CCH: Changhua Christian Hospital; HGT: Horizontal gene transfer; MDRAB: Multidrug-resistant *Acinetobacter baumannii*; PFGE: Pulsed-field gel electrophoresis; RIs: Resistant islands.

## Competing interests

The authors declare that they have no competing interests.

## Authors’ contributions

CHC and CCH designed and performed this study. CHC served the patients. CHC and CCH wrote the manuscript. Both authors read and approved the final manuscript.

## Supplementary Material

Additional file 1 **The literature summary of clinical multidrug-resistant *Acinetobacter baumannii *** **isolates between 2002 and 2004 in Taiwan.** Click here for file

Additional file 2 Infection rate of the hospital and the ICU during 2001–2004. Click here for file

Additional file 3 **PFGE fingerprints of 12 multi-resistant *A. baumannii *** **(MDRAB) isolates with resistance to ampicillin-sulbactam. ** Click here for file
