# Supplementary material for: Tracing the emergence of multidrug-resistant Acinetobacter baumannii in a Taiwanese hospital by evaluating the presence of integron gene intI1
Source: J Negat Results Biomed. 2014 Aug 14;13:15. doi: 10.1186/1477-5751-13-15 (PMC4155391; doi:10.1186/1477-5751-13-15)
Supplement: Additional file 1 — The literature summary of clinical multidrug-resistant Acinetobacter baumannii isolates between 2002 and 2004 in Taiwan. [file 1477-5751-13-15-S1.docx]

Additional file 1

Title of data : The literature summary of clinical multidrug-resistant *Acinetobacter baumannii* isolates between 2002 and 2004 in Taiwan

Description of data : We summary the reports of clinical multidrug-resistant *Acinetobacter baumannii* isolates between 2002 and 2004 in Taiwan

| Isolates/ Cases numbers | Comments | Resistant patterns | References |
| --- | --- | --- | --- |
| 203 isolates | The rapid emergence (from 0% before 1998 to 6.5% in 2000) of PDRAB was noted in a university hospital in Taiwan | All PDRAB | Hsueh PR.Emerg Infect Dis. 2002 ;8:827-32. |
| 58 isolates | a single strain of MDRAB was responsible for the prevalence of nosocomial infection amongst surgical patients, clearly differentiating this outbreak from the previous endemic situation | All MDRAB | Wu TL.J Hosp Infect. 2002 ;51:27-32. |
| 30 patients | The clinical outcome of patients with PDRAB bacteremia could be predicted by the MOD | 12 PDRAB patient | Kuo LC. J Formos Med Assoc 2003;102:601-606.; |
| 7 patients | PDRAB outbreak in surgical intensive care unit | All PDRAB | Wang SH. J Hosp Infect 2003;53:97-102.; |
| 6 infants | PDRAB outbreak in pediatric intensive care unit | NM | Huang YC. Pediatr Infect Dis J 2002;21:1105-1109 |
| 15 isolates | That PDRAB strain with an unusual phenotype could persist in humans for long periods and was widely disseminated throughout the hospital | All PDR-AB | Kuo LC. J Clin Microbiol. 2004;42:1759-63. |
| 111 isolates | There were no difference of IRAB between TSAR I (1998) and TSAR II (2000) isolates from 18 hospitals. | IRAB , 2 % ,in TSAR II (2000) | Lauderdale TL.Diagn Microbiol Infect Dis. 2004 ;48:211-9. |
| 15 isolates | Dissemination of a clone of unusual phenotype of PDRAB | All PDRAB baumannii | Kuo LC. J Clin Microbiol. 2004 ;42:1759-63. |

Notes : AB: *Acinetobacter baumannii ;* PDRAB: pandrug-resistant *Acinetobacter baumannii l* MDRAB: multidrug-resistant *Acinetobacter baumannii l* IRAB: imipenem resistant *Acinetobacter baumanniil ;* MDR Acb complex : multi-resistant *Acinetobacter calcoaceticus-A baumannii complex ;* EDRAB: extremely resistant *Acinetobacter baumannii ;* XDRAB: extensively drug-resistant *Acinetobacter baumannii ;* MOD: multiple organ dysfunction
