# Supplementary material for: Tracing the emergence of multidrug-resistant Acinetobacter baumannii in a Taiwanese hospital by evaluating the presence of integron gene intI1
Source: J Negat Results Biomed. 2014 Aug 14;13:15. doi: 10.1186/1477-5751-13-15 (PMC4155391; doi:10.1186/1477-5751-13-15)

Additional files 2

Title of data : The infection rate of the hospital and the ICU during 2001-2004

Description of data: The description of ICU infection rate, hospital infection rate, *A. baumanni* isolates, integrin carrying rate during the study years . The specifications of graphics in tables are the band for the *A. baumanni* isolates, dotted line with triangle for ICU infection rate, dotted line with square for hospital infection rate, solid line with round for integrin carrying rate.


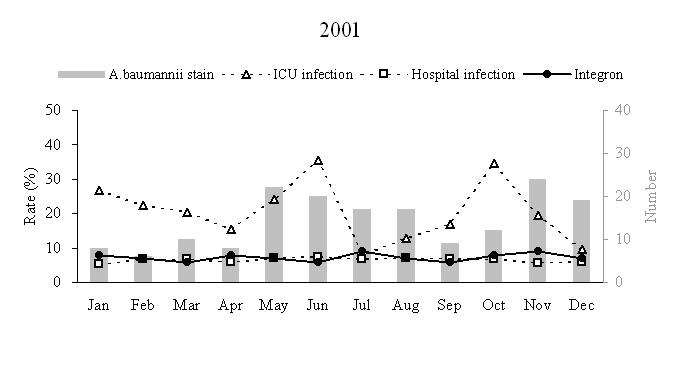


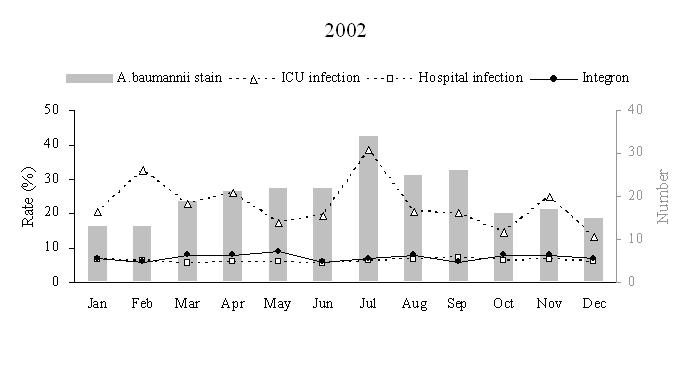


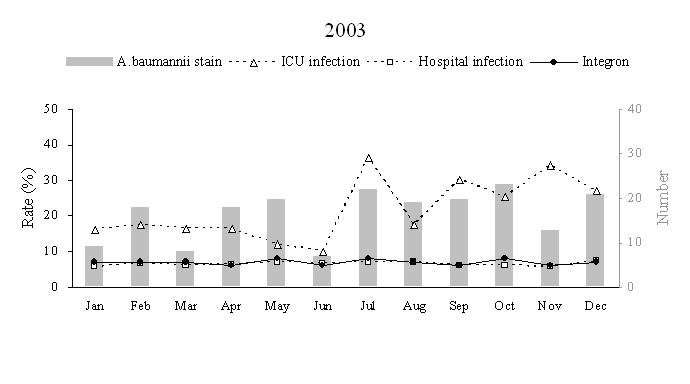

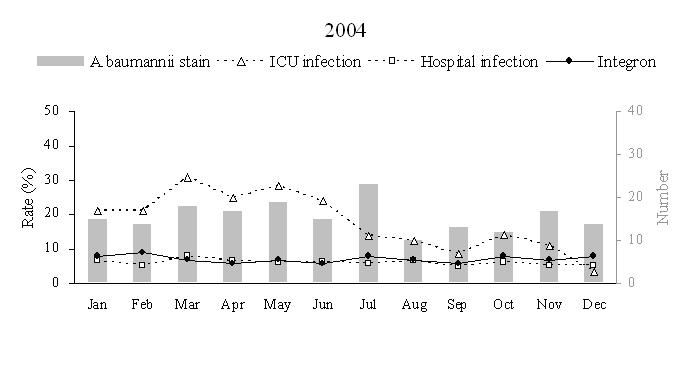

Supplement: Additional file 2 — Infection rate of the hospital and the ICU during 2001–2004. [file 1477-5751-13-15-S2.docx]
