# Supplementary material for: Tracing the emergence of multidrug-resistant Acinetobacter baumannii in a Taiwanese hospital by evaluating the presence of integron gene intI1
Source: J Negat Results Biomed. 2014 Aug 14;13:15. doi: 10.1186/1477-5751-13-15 (PMC4155391; doi:10.1186/1477-5751-13-15)

Additional file 3.

Title of data : PFGE fingerprints of 12 multi-resistant *A. baumannii* (MDRAB) isolates with resistance to ampicillin-sulbactam.

Description of data : PFGE fingerprints of 12 multi-resistant *A. baumannii* (MDRAB) isolates with resistance to ampicillin-sulbactam. Lane number of PFGE listed at Table 2 for the antibiotic susceptibility profiles; lanes M, molecular size markers (PFGE marker, *S. enterica* ser. *Braenderup* H9812).


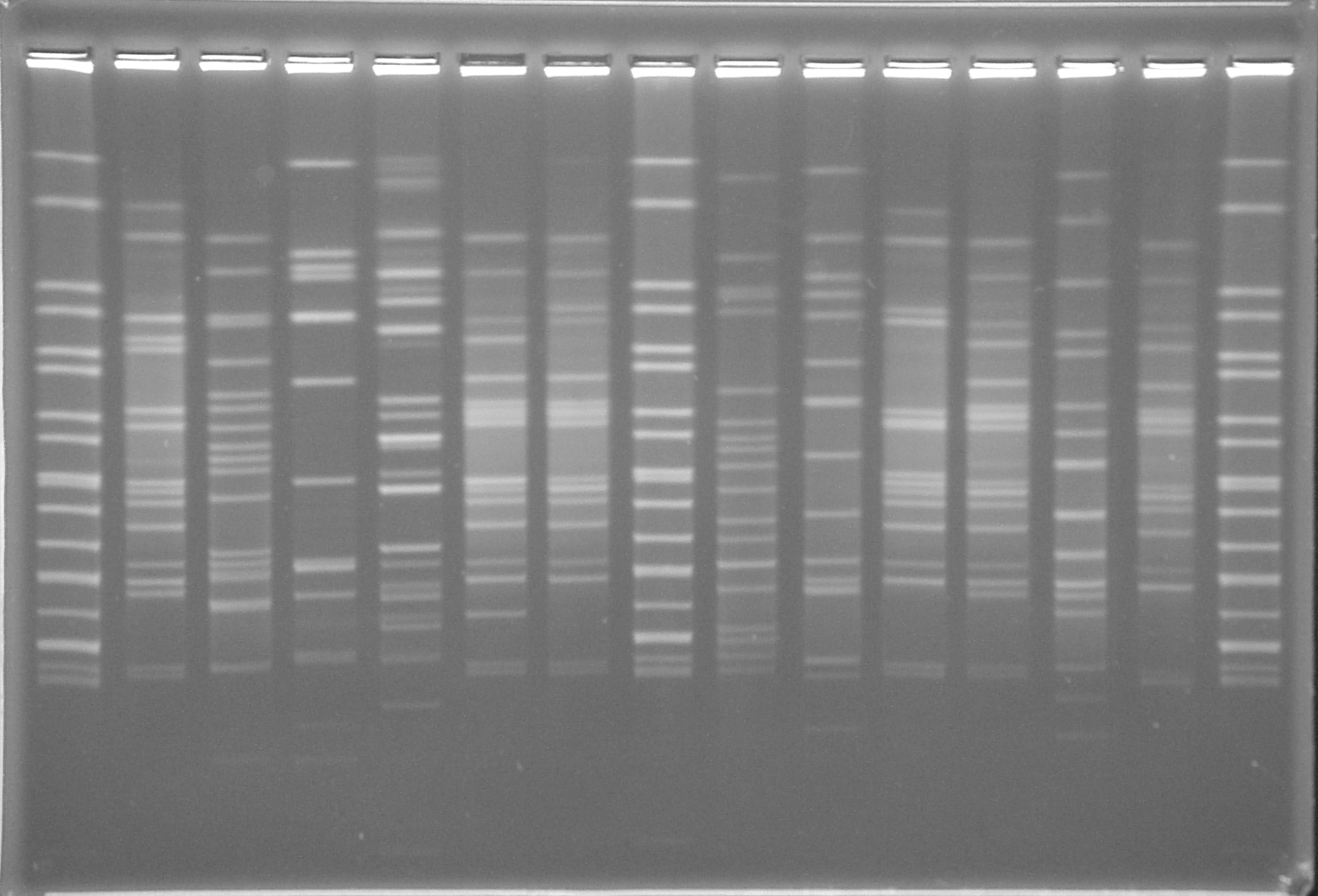


M P1 P2 P3 P4 P5 P6 M P7 P8 P9 P10 P11 P12 M


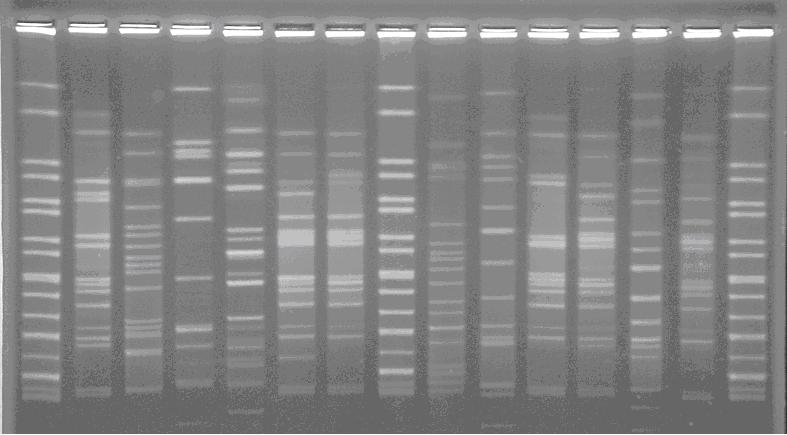

Supplement: Additional file 3 — PFGE fingerprints of 12 multi-resistant A. baumannii (MDRAB) isolates with resistance to ampicillin-sulbactam. [file 1477-5751-13-15-S3.docx]
